# Prevalence of methicillin-resistant *Staphylococcus aureus* in a tertiary hospital in Nepal

**DOI:** 10.5588/pha.21.0042

**Published:** 2021-11-01

**Authors:** P. Pradhan, P. Rajbhandari, S. B. Nagaraja, P. Shrestha, R. Grigoryan, S. Satyanarayana, H. Davtyan

**Affiliations:** 1 Department of Medical Microbiology and Immunology, Patan Academy of Health Sciences, Lalitpur, Nepal; 2 Department of Community Medicine, ESIC Medical College and Post Graduate Institute of Medical Science and Research, Bangalore, India; 3 World Health Organization Health Emergencies Programme, Kathmandu, Nepal; 4 Tuberculosis Research and Prevention Center, Yerevan, Armenia; 5 Centre for Operational Research, International Union Against Tuberculosis and Lung Disease, Paris, France

**Keywords:** prevalence, MRSA, antibiotic sensitivity pattern, treatment outcome, Nepal

## Abstract

**SETTING:**

Patan Hospital, Lalitpur, Nepal.

**OBJECTIVES:**

To describe 1) the prevalence of methicillin-resistant *Staphylococcus aureus* (MRSA) and its antibiotic sensitivity pattern; 2) the demographic and clinical characteristics associated with MRSA infections; and 3) the treatment outcomes of in-patients with MRSA infection among patients with *S. aureus* infection between January 2018 and December 2020.

**DESIGN:**

This was a cross-sectional study using electronic and paper-based hospital records of patients with *S. aureus* infection.

**RESULTS:**

Of the 1,804 patients with *S. aureus* infection*,* 1,027 patients (57%, 95% CI 55–59) had MRSA. The MRSA were susceptible to vancomycin (100%), linezolid (96%), doxycycline (96%), chloramphenicol (86%) and cotrimoxazole (70%), and resistant to erythromycin (68%), clindamycin (56%), gentamycin (58%), ciprofloxacin (92%) and ofloxacin (91%). The prevalence of MRSA was higher in 2019, among out-patients, and in respiratory samples, and lower in blood samples. Of the 142 in-patients with MRSA, 93% had a successful clinical outcome (cured/improved).

**CONCLUSION:**

More than 50% of patients with *S. aureus* infection had MRSA that were resistant to commonly available antibiotics. This calls for strengthening surveil-lance and good infection control practices in this hospital.

## Abstract (FR)

**LIEU:**

Hôpital de Patan, Lalitpur, Népal.

**OBJECTIFS:**

Décrire 1) la prévalence de *Staphylococcus aureus* résistant à la méticilline (MRSA) et son profil de sensibilité aux antibiotiques ; 2) les caractéristiques démographiques et cliniques associées aux infections à MRSA ; et 3) les résultats thérapeutiques des patients hospitalisés atteints d’infection à MRSA parmi ceux atteints d’infection à *S. aureus* de janvier 2018 à décembre 2020.

**MÉTHODE:**

Il s’agissait d’une étude transversale réalisée en utilisant les dossiers hospitaliers électroniques et papiers des patients atteints d’infection à *S. aureus*.

**RÉSULTATS:**

Sur les 1 804 patients atteints d’infection à *S. aureus,* 1 027 patients (57%, IC 95% 55-59) avaient un MRSA. Les MRSA étaient susceptibles à la vancomycine (100%), au linézolide (96%), à la doxycycline (96%), au chloramphénicol (86%) et au co-trimoxazole (70%), et résistants à l’érythromycine (68%), la clindamycine (56%), la gentamycine (58%), la ciprofloxacine (92%) et l’ofloxacine (91%). La prévalence des MRSA était plus élevée en 2019, parmi les patients ambulatoires, ainsi que dans les échantillons respiratoires. Elle était plus faible dans les échantillons sanguins. Sur les 142 patients hospitalisés avec MRSA, 93% ont connu un résultat clinique favorable (guérison/amélioration de l’état).

**CONCLUSION:**

Plus de 50% des patients atteints d’infection à *S. aureus* avaient un MRSA résistant aux antibiotiques habituellement disponibles. La surveillance et les pratiques de contrôle des infections doivent donc être renforcées dans cet hôpital.

Methicillin-resistant *Staphylococcus aureus* (MRSA) are genetic sub-variants of the *S. aureus* that are resistant to β-lactam antibiotics.^1^ Part of the normal bacterial flora of the skin and anterior nares in humans, *S. aureus* can cause different types of difficult-to-treat infections such as pyogenic skin infections, wound infections, bacteraemia, urinary tract infections and respiratory tract infections.^2^,^3^

Although MRSA infections can be acquired in community settings, it most commonly occurs in hospital settings, especially in those places where infection prevention and control measures are poorly implemented. These bacteria usually spread accidentally through direct contact with an infected wound or from the contaminated hands of healthcare providers. Also, people who are colonised with MRSA may not have signs of infection, and they can spread the bacteria to others.^4^

Mortality rates in patients with MRSA are known to be significantly higher than in those with methicillin-susceptible *S. aureus* (MSSA) infections (15.6% vs. 6.2%).^5^,^6^Addressing public health challenges associated with MRSA requires surveillance, good infection control practices, especially in hospitals, and access to life-saving antibiotics that are effective against MRSA.^7^

The prevalence of MRSA among those with *S. aureus* ranges from 13% to 74% in different parts of the world.^8^ According to the literature and data from various countries in the South-East Asian and the Western Pacific regions, MRSA prevalence among those with *S. aureus* infection is reported to be between 2% to 69%.^9^

In Nepal, a country in South Asia, MRSA prevalence among those with *S. aureus* infections ranges between 15% and 69% and varies widely across geographic locations.^10^Given the public health significance of MRSA infections, there is a need to monitor MRSA prevalence (as a part of surveillance for antimicrobial resistance [AMR]) in various settings, although the studies on MRSA or the results are not novel in our setting. Furthermore, there is minimal information on clinical management and treatment outcomes of inpatients with MRSA infection. We therefore conducted a hospital-based operational research study to determine the prevalence of MRSA in patients with *S. aureus* infection based on various documented demographic and clinical characteristics, antimicrobial sensitivity patterns to commonly used antibiotics and the treatment outcomes of inpatients with MRSA infection at Patan Hospital, a large tertiary care hospital in Lalitpur, Nepal.

The specific objectives were 1) to determine the number and proportion of specimens with MRSA among those who are culture-positive for *S. aureus* infections during January 2018 to December 2020; 2) to describe the drug resistance patterns of those with MRSA; and 3) to describe demographic, clinical characteristics and treatment outcomes of in-patients with MRSA infection.

## METHODS

### Study design

This is a descriptive cross-sectional study involving a review of secondary data from electronic databases and paper-based patient records at Patan Hospital.

### Setting

Nepal, a low-income, landlocked country bordering China and India, consists of seven provinces with an estimated population of 30.2 million.^11^Healthcare services are offered by both public and private providers. Infectious diseases remain among the top causes of death. As of 2017, these are highly prevalent.^12^Antibiotics are sold over the counter and are readily available to the general public.^13^

However, the importance of the problem has been recognised, and an integrated response to combat AMR has been included in the Nepal Health Sector Strategy Plan. The National Action Plan (NAP) to fight AMR is still under development and is being adapted from the five strategic objectives (awareness, surveillance, infection prevention and control, antimicrobial usage, and research and innovation) of the global action plan for AMR. The incorporation of AMR activities in the existing programme is currently very limited, or none in some cases.^14^

Nepal was enrolled in the Global Antimicrobial Surveillance System (GLASS) in 2018, with 21 sentinel laboratories performing antimicrobial susceptibility testing (AST). These sites perform routine culture and AST for different types of specimens. The test result data are recorded in local databases and reported to the National Public Health Laboratory (NPHL). NPHL acts as a national coordinating centre by validating, combining and uploading all data to GLASS annually, and provides external quality control to participating laboratories. In 2018, only 15 (out of 21) provided data for GLASS, and the microbiology laboratory at Patan Hospital was one of those.^15^

Patan Hospital is located in the Lalitpur District of Nepal. It is a 650-bed, tertiary-care teaching hospital attached to the Patan Academy of Health Sciences (PAHS). As one of the largest hospitals in Nepal, it provides quality care to nearly 320,000 outpatients and 20,000 in-patients per year.

The hospital’s microbiology laboratory, one of the sentinel laboratories for AMR surveillance, has internal and external quality control programmes operated by the NPHL and Christian Medical College, Vellore, India. Blood specimens were collected in BD BACTECTM (BD, Franklin Lakes, NJ, USA) aerobic bottles and incubated until bacterial growth, which was detected using BD BACETCTM FX blood culture system. Specimens with growth were inoculated onto blood agar, chocolate agar and MacConkey agar. Other biological specimens such as pus, respiratory secretions, urine samples were inoculated onto blood, chocolate or MacConkey agar depending on the type of specimen. Culture samples were aerobically incubated at 37°C for 24–48 h. *S. aureus* were identified using Gram’s stain, catalase and coagulase tests. AST was performed using the modified Kirby Bauer disk diffusion method as recommended in the Clinical and Laboratory Standards Institute (CLSI; Wayne, PA, USA) guidelines.^16^MRSA was detected using Cefoxitin disc (30 μg). Results of the performed tests were entered into the electronic hospital information system.

### Study population and study period

For objectives 1 and 2, we included all patients who submitted biological specimens for culture and AST from January 2018 to December 2020 and were positive for *S. aureus*. These patients were selected by reviewing the electronic hospital information system. From this patient population, we selected in-patients whose biological specimens yielded MRSA for objective 3.

### Data sources and collection

Patients’ demographic profiles and laboratory results were obtained from the electronic database of the hospital information system and entered into a Microsoft Office Excel spreadsheet (Microsoft, Redmond, WA, USA). A unique patient encounter number (EncID) recorded in the electronic database was used to trace the hospital number of admitted patients. A permanent hospital number was assigned to patients on admission to the hospital. This permanent number was used to trace the inpatient’s record files. Data on clinical characteristics and antibiotics usage profile were retrieved from these files held at the archives of Patan Hospital. Data were first entered in a proforma, and these data were later entered into EpiData software v3.1 (EpiData Association, Odense, Denmark).

### Data analysis

Data entered into EpiData were exported and analysed using the statistical software Stata v15.1 (StataCorp, College Station, Tx, USA). AWaRe classification (‘Access’, ‘Watch’ and ‘Reserve’ group of antibiotics) was used to group antibiotics for AST.^17^ Data are summarised using frequencies and percentages. We used bivariable binomial log models to study the association between demographic and clinical factors with demographic and clinical characteristics. These associations are presented as prevalence ratios; *P*<0.05 was considered statistically significant.

### Ethics considerations

The study obtained ethics approval from the Institutional Review Committee of Patan Academy of Health Sciences, Lalitpur, Nepal (Ref: bss2102231481/Date: 021-02-23) and from the Ethics Advisory Group of the International Union Against Tuberculosis and Lung Disease, Paris, France (EAG number: 04/20, Date 2020-02-05). As this study involved analysing of retrospective data from routine records, the need for informed consent was waived; data confidentiality was maintained throughout the study.

## RESULTS

Of the 114,137 samples received for culture between 2018 and 2020, 1,804 showed *S. aureus* growth. The antibiotics used for AST, the number of samples tested for each antibiotic and the susceptibility/resistance pattern is given in Table 1.Out of the 13 antibiotics, all samples were tested for resistance to oxacillin. The prevalence of MRSA (defined as resistance to oxacillin) among those with *S. aureus* was found to be 57% (95% confidence interval [CI] 55–59).Other than oxacillin, 98% of the *S. aureus* samples were tested for seven other antibiotics: cotrimoxazole, clindamycin, gentamycin and chloramphenicol belonging to the Access group of antibiotics; and ciprofloxacin, ofloxacin and erythromycin belonging to the ‘Watch’ group of antibiotics. For these seven antibiotics, resistance levels ranged from 22% for cotrimoxazole to 72% for ciprofloxacin. About 24% of the *S. aureus* isolates that were multidrug-resistant were tested for linezolid, the antibiotic belonging to the ‘Reserve’ group; 3% of the samples were found to be resistant to it.

**TABLE 1:** Results of antibiotic susceptibility testing of Staphylococcus aureus (n=1804) isolated from biological samples of patients submitted to the microbiology laboratory at Patan Hospital, Lalitpur, Nepal, 2018–2020

Antibiotics	AWaRe classification	Samples tested		Susceptible		Resistant		Intermediate	
			
		*n*	(%)	*n*	(%)	*n*	(%)	*n*	(%)
Oxacillin	Access	1,804	(100)	777	(43)	1,027	(57)	0	(0)
Cotrimoxazole	Access	1,764	(98)	1,246	(71)	383	(22)	135	(8)
Clindamycin	Access	1,795	(99)	881	(49)	734	(41)	180	(10)
Gentamicin	Access	1,768	(98)	899	(51)	685	(39)	184	(10)
Chloramphenicol	Access	1,790	(99)	1,590	(89)	54	(3)	146	(8)
Doxycycline	Access	965	(53)	934	(97)	20	(2)	11	(1)
Nitrofurantoin	Access	7	(1)	7	(100)	0	(0)	0	(0)
Ciprofloxacin	Watch	1,795	(99)	436	(24)	1,286	(72)	73	(4)
Ofloxacin	Watch	1,778	(99)	510	(29)	1,252	(70)	16	(1)
Erythromycin	Watch	1,791	(99)	572	(32)	964	(54)	255	(14)
Vancomycin	Watch	423	(23)	423	(100)	0	(0)	0	(0)
Azithromycin	Watch	82	(5)	13	(16)	69	(84)	0	(0)
Linezolid	Reserve	433	(24)	418	(97)	15	(3)	0	(0)

AWaRe = ‘Access’, ‘Watch’ and ‘Reserve’ group of antibiotics.

The AST pattern of MRSA samples is given in Table 2.Although not all samples were tested for antibiotic resistance, 100% of those tested were susceptible to vancomycin, 97% to linezolid and 96% to doxycycline. In addition to these, MRSA were also susceptible to chloramphenicol (89%) and cotrimoxazole (71%). MRSA were highly resistant (>90% of the samples) to ciprofloxacin and ofloxacin.

**TABLE 2:** Results of antibiotic susceptibility testing of methicillin-resistant Staphylococcus aureus (n=1027) isolated from biological samples of patients submitted to the microbiology laboratory at Patan Hospital, Lalitpur, Nepal, 2018–2020

Antibiotics	AWaRe classification of antibiotics	Samples tested		Sensitive		Resistant		Intermediate	
			
		*n*	(%)	*n*	(%)	*n*	(%)	*n*	(%)
Cotrimoxazole	Access	1,002	(98)	698	(70)	207	(21)	97	(10)
Clindamycin	Access	1,021	(99)	369	(36)	570	(56)	82	(8)
Gentamicin	Access	1,005	(98)	257	(25)	578	(58)	170	(17)
Chloramphenicol	Access	1,018	(99)	879	(86)	39	(4)	100	(10)
Doxycycline	Access	574	(56)	550	(96)	18	(3)	6	(1)
Nitrofurantoin	Access	5	(1)	5	(100)	0	(0)	0	(0)
Ciprofloxacin	Watch	1,021	(99)	72	(7)	936	(92)	13	(1)
Ofloxacin	Watch	1,014	(99)	87	(8)	918	(91)	9	(1)
Erythromycin	Watch	1,021	(99)	217	(21)	696	(68)	108	(11)
Vancomycin	Watch	263	(26)	263	(100)	0	(0)	0	(0)
Azithromycin	Watch	75	(7)	7	(9)	68	(91)	0	(0)
Linezolid	Reserve	267	(26)	258	(97)	9	(3)	0	(0)

AWaRe = ‘Access’, ‘Watch’ and ‘Reserve’ group of antibiotics.

The demographic and clinical characteristics of the patients from whom *S. aureus* and MRSA were isolated are given in Table 3. In those with *S. aureus* infection, the prevalence of MRSA was similar in both the sexes in different age groups. Almost all samples from in-patients had MRSA when compared to ~50% among samples from outpatients. The prevalence of MRSA was higher in 2019 than in 2018 (prevalence ratio [PR] 1.10, 95% CI 1.00–1.20], in outpatients than in-patients (PR 2.38, 95% CI 2.05–2.75) and among *S. aureus* isolated from respiratory specimens when compared to pus (PR1.33, 95% CI 1.10–1.62). MRSA prevalence was lower among *S. aureus* isolated from blood when compared to pus (PR 0.80, 95% CI 0.65–0.98).

**TABLE 3:** Demographic and clinical characteristics of patients with MRSA infection among those with Staphylococcus aureus infection isolated from biological samples of patients submitted to the microbiology laboratory at Patan Hospital, Lalitpur, Nepal, 2018–2020

Demographic and clinical characteristics	Individuals with SA *n*	Individuals with MRSA		Prevalence ratio (95% CI)

		*n*	(%)	
Total	1804	1027	(56.9)	
Year				
2018	739	409	(55.3)	Reference
2019	722	440	(60.9)	1.10 (1.00–1.20)^*^
2020	343	178	(51.9)	0.93 (0.83–1.05)
Sex				
Female	916	530	(57.9)	Reference
Male	888	497	(56.0)	0.96 (0.89–1.04)
Age group, years				
<1	158	100	(63.3)	1.09 (0.95–1.25)
1–5	197	123	(62.6)	1.08 (0.95–1.22)
6–18	343	180	(52.5)	0.90 (0.80–1.02)
19–35	650	377	(57.9)	Reference
36–50	210	117	(55.7)	0.96 (0.83–1.10)
51–65	139	72	(51.8)	0.89 (0.75–1.06)
>65	107	58	(54.2)	0.93 (0.77–1.12)
Hospitalisation				
Inpatients	514	142	(27.6)	Reference
Outpatients	1202	796	(66.2)	2.38 (2.05–2.75)^*^
Unknown	88	88	(100)	1 (NE)
Specimen				
Blood	114	53	(46.5)	0.80 (0.65–0.98)^*^
Body fluid	8	4	(50.0)	0.86 (0.43–1.73)
Body swab	65	32	(49.2)	0.85 (0.66–1.09)
Catheter tips	3	3	(100.0)	1 (NE)
Genital swab	9	2	(22.2)	0.38 (0.11–1.30)
Pus	1560	902	(57.8)	Reference
Respiratory sample	31	24	(77.4)	1.33 (1.10–1.62)^*^
Tissue	1	1	(100.0)	1 (NE)^†^
Urine	13	6	(46.2)	0.79 (0.44–1.43)

^*^ *P* < 0.05.

MRSA = methicillin-resistant *Staphylococcus aureus*; CI = confidence interval; NE = not estimated.

The demographic and clinical characteristics of these 142 in-patients with MRSA infection and their treatment outcomes are given in Table 4. Although not all patients in this group were tested to the various groups of antibiotics, the MRSA was susceptible to vancomycin (100%), linezolid (95%), doxycycline (94%), chloramphenicol (86%) and cotrimoxazole (72%) in those tested. Based on the laboratory AST report, the initial antibiotics used for treatment in this group were changed in 74% of cases. The median duration of hospitalisation of these in-patients was 7 days (interquartile range: 5–13), and 93% had a successful treatment outcome (cured/improved).

**TABLE 4:** Characteristics and treatment outcomes of inpatients (n=142) having infection with MRSA at Patan Hospital, Lalitpur, Nepal, 2018–2020

Demographic and clinical characteristics	Individuals with MRSA	

	*n*	(%)
Year		
2018	40	(28)
2019	75	(53)
2020	27	(19)
Sex		
Female	68	(48)
Male	74	(52)
Age group, years		
<1	17	(12)
1–5	20	(14)
6–18	22	(15)
19–35	35	(25)
36–50	17	(12)
51–65	13	(9)
>65	18	(13)
Specimen		
Blood	10	(7)
Body swab	2	(1)
Pus	123	(87)
Respiratory sample	7	(5)
Sensitive to antibiotic^*^		
Cotrimoxazole	101	(72)
Gentamicin	43	(31)
Ciprofloxacin	6	(4)
Ofloxacin	6	(4)
Chloramphenicol	122	(86)
Erythromycin	37	(26)
Clindamycin	53	(37)
Doxycycline	89	(94)
Vancomycin	42	(100)
Azithromycin	2	(22)
Linezolid	40	(95)
New antibiotic introduced after laboratory drug-susceptible report		
Yes	105	(74)
No	37	(26)
Treatment outcome		
Cured	9	(6)
Improved	124	(87)
No change	1	(1)
Discharged against medical advice	5	(4)
Unknown	3	(2)

^*^ Not all samples were tested for antibiotic sensitivity; the proportion shown to be susceptible here is among those tested for antibiotic susceptibility.

MRSA = methicillin-resistant *Staphylococcus aureus*.

## DISCUSSION

This is the first study on AST from Patan Hospital; we report that more than half of the patients (1,804 biological samples with *S. aureus* infection) had MRSA that was resistant to several ‘Access’ and ‘Watch’ group antibiotics, and more than 90% of the in-patients with MRSA infection had successful treatment outcomes. Study findings provide the following perspectives on *S. aureus* and MRSA infection in our hospital setting.

First, the prevalence of MRSA among those with *S. aureus* was 57% in our study, which is higher than that of a study conducted by Kshetry et al., which reported the prevalence of MRSA to be 37.6%.^18^ In a similar study conducted by Sapkota et al., MRSA prevalence was found to be 70.6%, which was higher than that of our study.^19^ Other studies conducted at different time points in the same geographical area have shown relatively lower prevalences of MRSA than that of our study.^10^ As our hospital is a tertiary referral hospital, prevalence in our setting may have been higher due to the selective referral of patients who had not responded to antibiotics, or due to rising MRSA infections in the community. Increasing levels of MRSA infection in the community is a cause for concern and calls for strengthening infection prevention and control measures and improved access to second-line antibiotics to treat such infections. In our study, most MRSA were sensitive to chloramphenicol (89%) and cotrimoxazole (71%) in the ‘Access’ group of antibiotics, and this is a positive sign, as both these antibiotics are easily accessible and cheap. Most MRSA infections in our setting may therefore not need costly second-line antibiotics (vancomycin, linezolid) for treatment.

Second, the prevalence of MRSA was found to be higher in 2019 than in 2018. The decline in the number of samples in 2020 may have been due to the reduction in hospital visits because of COVID-19-induced travel restrictions. Furthermore, MRSA prevalence was higher among respiratory samples than in pus samples, and lower among blood samples. Previous studies have shown that a higher prevalence of MRSA is associated with sex (higher among males than in females),^20^ age (higher among the elderly than in the young),^20^ and among in-patients than in out-patients.^20^ We did not find such associations in our study despite the large sample size.

Third, despite fewer MRSA in-patient records studied for treatment outcomes, we noted that treatment outcomes were good in more than 93% of the patients, with a 0% mortality rate. This contrasts with the studies conducted elsewhere, where mortality rates have been up to 15% in those with MRSA infection.^5^,^6^Apart from possible selection bias, we believe that the factor contributing to high treatment success levels was perhaps MRSA susceptibility to a large number of commonly available antibiotics, such as chloramphenicol and cotrimoxazole. However, because of the low proportion of in-patients studied, our study findings on treatment outcomes may not be generalisable to other patients whose records were not studied. This is an area for future prospective research.

The major strengths of the study were as follows: 1) we reviewed a large dataset of *S. aureus* samples; 100% of these samples assessed for oxacillin resistance in a microbiology laboratory with good laboratory practices (in accordance with CLSI guidelines). We therefore believe that our study provides a reliable estimate of the resistance of *S. aureus* to various antibiotics and MRSA prevalence in our hospital setting. 2) We conducted the study using routine hospital data; therefore, the study reflects the ground reality under routine conditions. The major limitations of the study are as follows: 1) we did not have information on the referral practices of clinicians’ bacterial culture and AST. Therefore, we do not know whether all patients likely to have *S. aureus* underwent bacterial culture and AST. This limits our ability to generalise the study findings beyond our study population. 2) As not all samples of MSSA and MRSA were tested to all antibiotics, we are unable to assess multidrug resistance and compare the multidrug resistance profiles for MSSA and MRSA. 3) Information on MRSA treated in the outpatient department was not available in the hospital records; future research into treatment outcomes of such patients is warranted.

In conclusion, more than half of the patients with *S. aureus* infection at Patan Hospital had MRSA. The MRSA prevalence among *S. aureus* infection was higher in 2019, and in respiratory samples, but lower in blood samples. The most common antibiotics to which MRSA was susceptible were vancomycin, linezolid, doxyclinine, chloramphenicol and cotrimoxazole; treatment outcomes of in-patients with MRSA were good, with more than 90% having a favourable outcome.
